# Power imbalance in hospital dual management system and its impact on turnover

**DOI:** 10.3389/fpubh.2024.1458411

**Published:** 2024-09-25

**Authors:** Manli Zhang, Qianqian Guo, Janak L. Pathak, Vian Ou, Le Li, Virginia Trigo

**Affiliations:** ^1^School and Hospital of Stomatology, Guangdong Engineering Research Center of Oral Restoration and Reconstruction, Guangzhou Medical University, Guangzhou, China; ^2^School of Health Management, Southern Medical University, Guangzhou, China; ^3^ISCTE University Institute of Lisbon, Lisbon, Portugal

**Keywords:** hospital dual management, power imbalance, turnover, grounded theory, empirical research

## Abstract

**Introduction:**

With the deepening of the healthcare reform in China, the competition in the sector is becoming fiercer leading to several unintended consequences such as talent turnover, which highly affect hospital development. Taking the case of a specialized university-affiliated stomatology hospital in Guangzhou, this thesis aims to analyze the main factors that contribute to talent turnover in this type of hospitals. The loss of talents in the field of stomatology is not unique to China and represents a significant problem in both developed and developing countries.

**Methods:**

The data for the study were collected from *post facto* live interviews conducted with 21 resigners to understand their feelings and motivations after the passage of time. The data were coded and concepts refined using MAXQDA software to form 18 sub-categories and six aggregate dimensions leading to the construction of two theoretical models.

**Results:**

The balance of power embedded in the dual leadership system characteristic of Chinese organizations and corresponding leadership style and behavior, deeply affect the turnover of talents. The more unbalanced the power, the greater the impact on turnover.

**Conclusion:**

Based on the situation studied, a power imbalance allowed for individual behaviors that determined the development of the hospital and directly affected institutional fairness, culture, working atmosphere and ultimately led to turnover.

## Introduction

In recent years, the relationship between doctors and patients has become increasingly tense, with frequent incidents of medical disputes, resulting in a declining sense of professional security among doctors. The continuous departure of medical personnel has further strained the already scarce human resources in health care, and has become one of the important factors restricting the development of health care in less developed areas (1). Excessive brain drain will lead to imbalance in the echelon structure, increase the replacement cost, and affect the level and quality of medical services. With the full implementation of hierarchical diagnosis and treatment and multi-practice system, the channels for medical talents to choose employment and career are more open and diversified, and the free flow of talents has become a “new normal” in today’s society (2). If the medical industry wants to be invincible in the fierce competition, it all depends on whether the hospital has a highly competitive professional talent and an efficient and reasonable medical talent ladder. University-affiliated hospitals are affiliated with universities and are responsible for medical care, teaching and research (3). University-affiliated specialty hospitals are generally the hospitals with the highest level of specialty care in the region, and are a talent-intensive organization. University-affiliated specialized stomatology hospitals are generally managed as stomatology hospitals, schools of stomatology, and institutes of oral diseases, and are responsible for stomatology work, teaching, postgraduate training, and scientific research. This study takes university-affiliated stomatology specialty hospitals as the research object, and combines relevant actual cases to conduct a grounded theoretical and empirical study.

Research about turnover started to appear more consistently in the 1960s and 1970s with its determinants being the most concerned topic (4), specifically the factors leading to voluntary turnover. Early research by March (1958) indicated two factors (5)—turnover intention and the ease of turnover—followed by the psychological changes proposed by Mobley (6) and the unfolding model of voluntary turnover proposed by Lee (1996, 1999), along with social capital theory or cultural factors (7). Generally speaking, scholars have achieved remarkable achievements in the field although there is still not a consistent agreement about why employees leave the organization voluntarily and the discussion continues.

The study of turnover started late in China and is still a relatively weak field. Chinese scholars have mainly analyzed the issue from quantitative and qualitative aspects. In terms of quantitative studies, scholars discuss the issues from two perspectives: one is the study of the effect of demographic and career variables on turnover, verified in specific groups; the other is to explore the applicability of Western models among Chinese employees, and then revise the model based on research results. In turn, qualitative research in China reviews the mainstream models and theories done by Western scholars and puts forward relevant suggestions and measures. Chinese scholars’ research on turnover was mainly focused on reviewing mainstream Western turnover models. After 2005, Chinese scholars began to discuss the applicability of mainstream Western turnover models to Chinese organizations and to conduct endogenous empirical studies.

Grounded theory, as an inductive qualitative method, allows researchers to identify the main concerns of a group of subjects and the behaviors used to address them (8). It enables researchers to have a deeper understanding not only of the main concerns but also of accumulated concerns experienced by participants and possibly to develop a theory that captures their behavior (8).

The General Office of the CPC Central Committee issued the Opinions on Strengthening Party Building in Public Hospitals in 2018 (9). The opinions made it clear that the president of a public hospital takes responsibility under the leadership of the Party committee, and, in the case of a grade 2 hospital or above, he or she cannot concurrently serve as the Party secretary. To integrate the leadership of the Party in hospital governance, the president and the secretary of the Party committee shall be two people respectively, one in charge of hospital administration and the other in charge of hospital Party organization leadership.

If we compare the hospital to a ship, the Party committee is the helmsman of the ship, and the president sails the ship. The division of labor is clear: the helmsman directs the ship and keeps it on the right course; the rowers are responsible for keeping the boat powered. The president’s responsibility system under the leadership of the Party committee should be well implemented so that the Party committee and the president cooperate closely and complement each other. On the contrary, if any one of the parts is missing, the ship will fail to sail in the right direction. This system highlights the centralized discussion by the Party committee and the combination of collective leadership and individual responsibility, which avoids personal preference and favors “one word” in the decision-making of major issues.

Based on reviewing the existing qualitative interview and grounded theory literature on the influencing factors of employee turnover intention in the medical and health industry, this paper aims at understanding and uncover the causes of brain drain and find the related concepts and categories to construct a theoretical model.

## Methods

This study takes the case of one specific hospital in Guangzhou to examine the research problem: the brain drain that is afflicting Chinese hospitals in general, and Chinese stomatology hospitals in particular. The study follows a case study approach and adopts qualitative methods to analyze and explore the influencing factors that affect the brain drain in the hospital. Data were collected from interviews and analyzed with the help of grounded theory. Twenty one interviews were selected among the 237 leavers who quit the hospital from 2013 to 2020. They are the talents who are the subject of this thesis that is professionals and managers with MSc or Ph.D. degrees from different positions, titles, and ranks.

### The research object

Since the reform and opening up of China, with the growing demand for oral healthcare services among Chinese nationals, dental hospitals across China have developed rapidly. The mode of coexistence of public stomatology hospitals, Chinese-foreign joint venture hospitals, private hospitals and individual medical institutions with various modes of operation has gradually taken shape, and this mode is dominated by public stomatology hospitals. Public stomatology hospitals, with public welfare as their purpose, are the main concentration of medical resources such as medical technology, equipment, and talents, and have strong comprehensive advantages in the treatment of difficult diseases, medical research, and education and training, and are able to provide a full range of medical services.

The hospital under study is a municipal tertiary-level A-class dental specialty hospital integrating medical treatment, teaching, research, prevention and health care. The hospital is ranked in the upper-middle level in the 2023 Chinese National Performance Assessment for Tertiary Public Hospitals, which is universally representative.

The hospital lost a total of 237 people in 8 years from January 2013 to December 2020, with senior titles, formal staffing and young and middle-aged talents as the main targets of loss, and the talent loss showed an increasingly serious trend.

### Research process

As this study seeks to understand the full range of factors that influence dental talent turnover and the grounded theory provides a good method to explore the field of healthcare, this thesis follows this approach. In a grounded theory approach, the researcher first asks a specific question, which leads to the development of other concepts in the research topic. Specifically, the researcher asked dental professionals how they felt about leaving, and then, building on their answers, came up with more ideas and topics. This means that research questions are asked and answered with a focus on specific situations of specific people at specific times and places, based on participant data.

### Research design

The subject of this study lost 237 talents during the period from January 2013 to December 2020. As this study focuses on the departure factors of high-level talents, the researcher selected the exit interview data of 83 leavers, that is all those with MSc degrees or above among the 237 professionals who left the hospital during that period.

To gain a deeper understanding of the real reasons why high-level talents leave their jobs, in-depth interviews need to be carried out with the goal of theoretical saturation. In 2022, a second round of face-to-face interviews was conducted with 21 high-level talents out of the initial group of 83. Based on the principle of information saturation, the richer and more diverse the information is evaluated, the more information each person represents, and the fewer in-depth interviewees are needed (10). Theoretical saturation is reached when every code in the group is observed at least once (11). Similarly, qualitative inquiries are sufficient to meet the needs of the research topic through generalization as long as the “maximum variance information saturation”is relatively achieved (12).

The study conducted in-depth interviews with people in different positions, job titles, and ranks to achieve information saturation after initial analysis of textual information from exit interviews with 83 people. The final number of in-depth interviews was 21.

The in-depth interview data of 21 people were used as the core data of this study for coding analysis and theoretical exploration research. The interviews were conducted from 5 December to 30 December 2021, at the case hospital. The average age of the interviewees was 42.6 (age span of 32–56) and some of the interviewees had been working in the hospital for several years, especially some who had been in management positions for up to 10 years and had a better understanding of the deeper issues in hospital management. This paper used MAXQDA20.0 for coding and qualitative data management and analysis of the collected interview records and materials. The data obtained from the interviews were loaded into MAXQDA software.

The first step was open coding to conceptualize and scope of the data to obtain the initial concepts or categories; the second step was axial coding to classify, compare and extract different concepts or categories so as to obtain the main themes. In the third step, selective coding was carried out to extract the core categories that could unify all concepts by continuously comparing the relationships between different main concepts or categories.

## Analysis of the coding process and results

**Figure 1:**
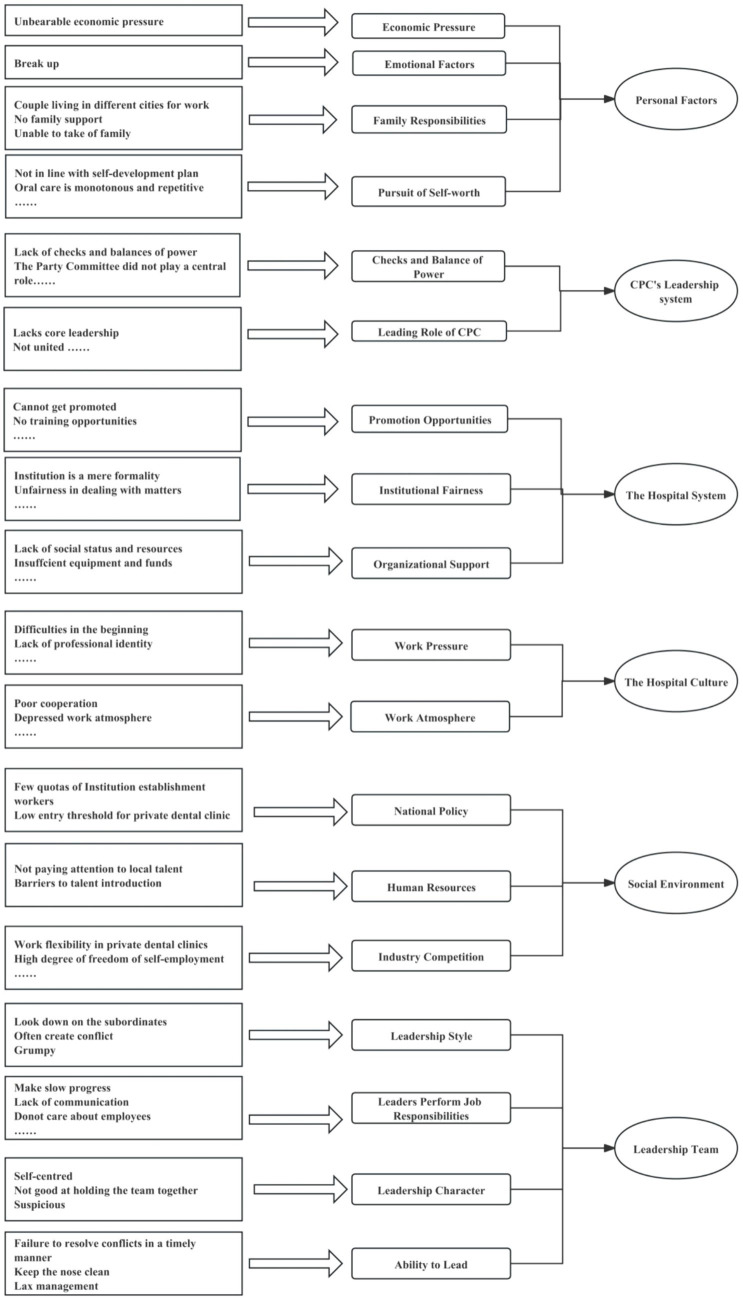
Three-level coding chart for 21-person in-depth interview data.

**Figure 2:**
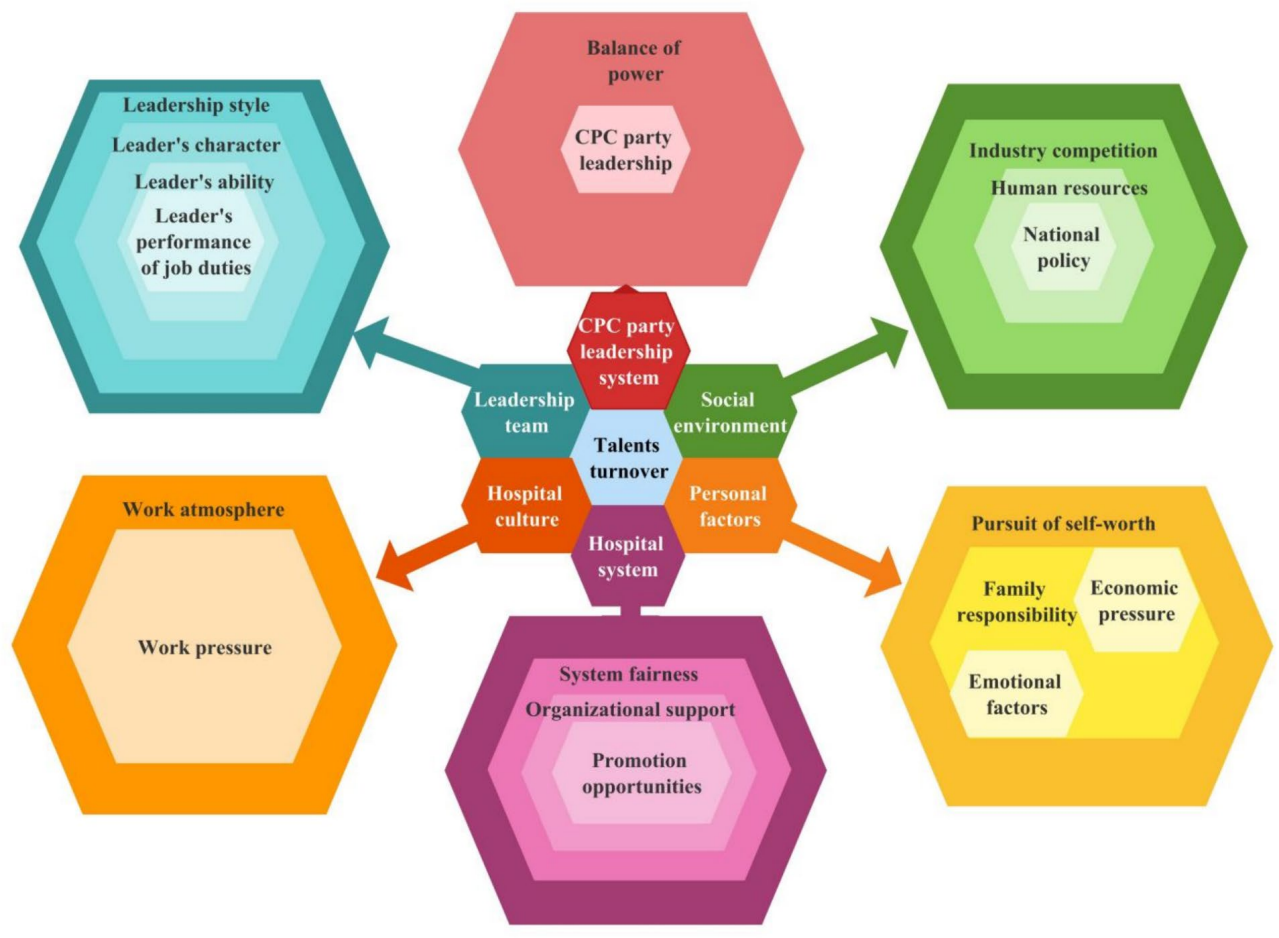
Chart of axial and selective coding for the 21 in-depth interviews.

The interview notes were arranged and imported into MAXQDA software for coding resulting in a total of 113 open coding that were collated and refined to form 18 sub-categories. At the end of the three levels of coding, six categories were finally formed (see Figures 1, 2). These categories all point to the factors of brain drain that is the subject of this research as it is detailed below.

### Category 1: personal factors

**Figure 3:**
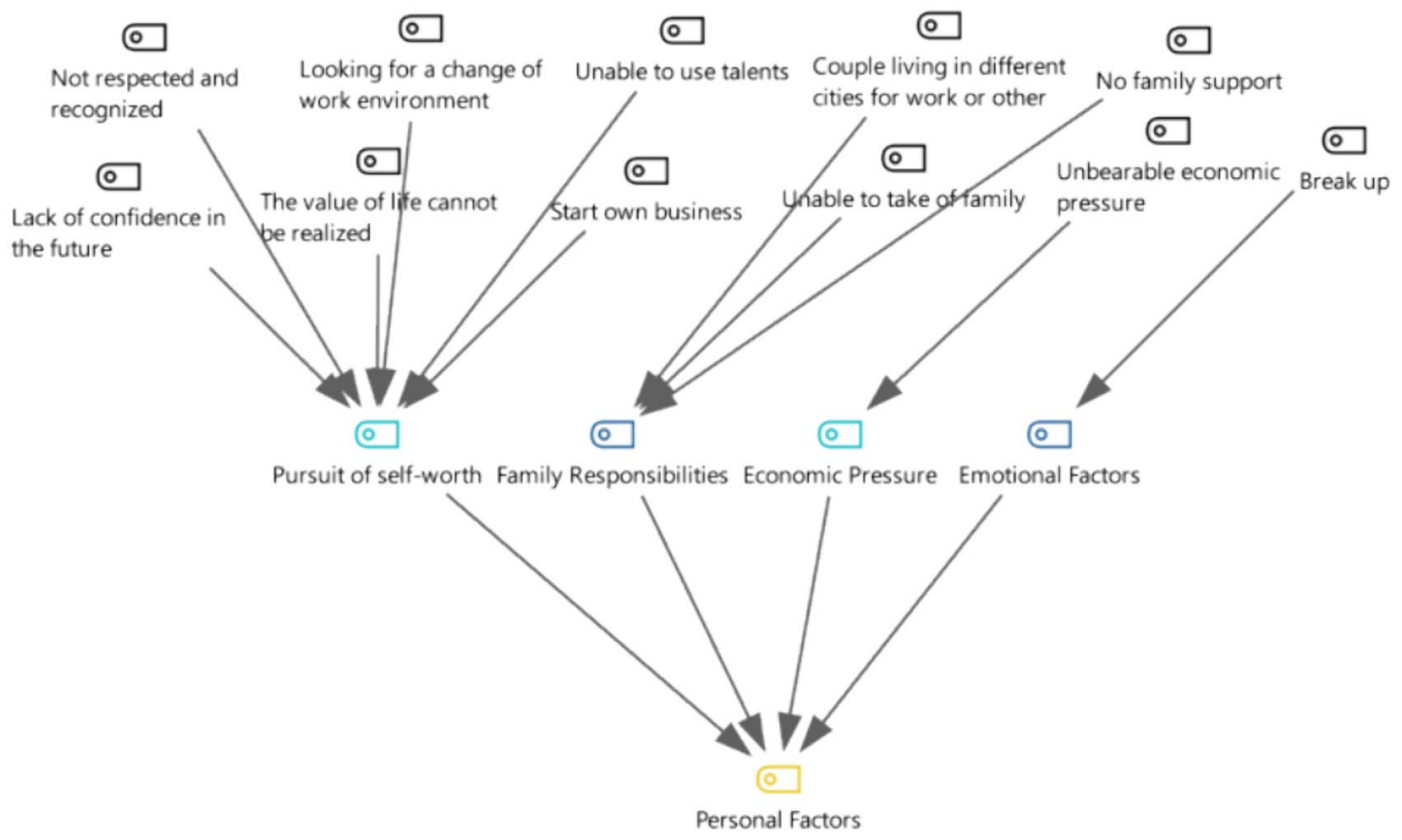
Three-level coding chart for the personal factors category.

Analyzing the data, the four categories “financial stress,” “emotional factors,” “family responsibilities” and “pursuit of self-worth” were aggregated into the main category (theme) “personal factors” (see Figure 3). “Financial pressures,” “emotional factors,” and “family responsibilities” are the result of individual decisions and events that surround the individual. The “pursuit of self-worth” is related to self-awareness, which is the basic intrinsic drive for self-actualization in most people (13).

**Figure 4:**
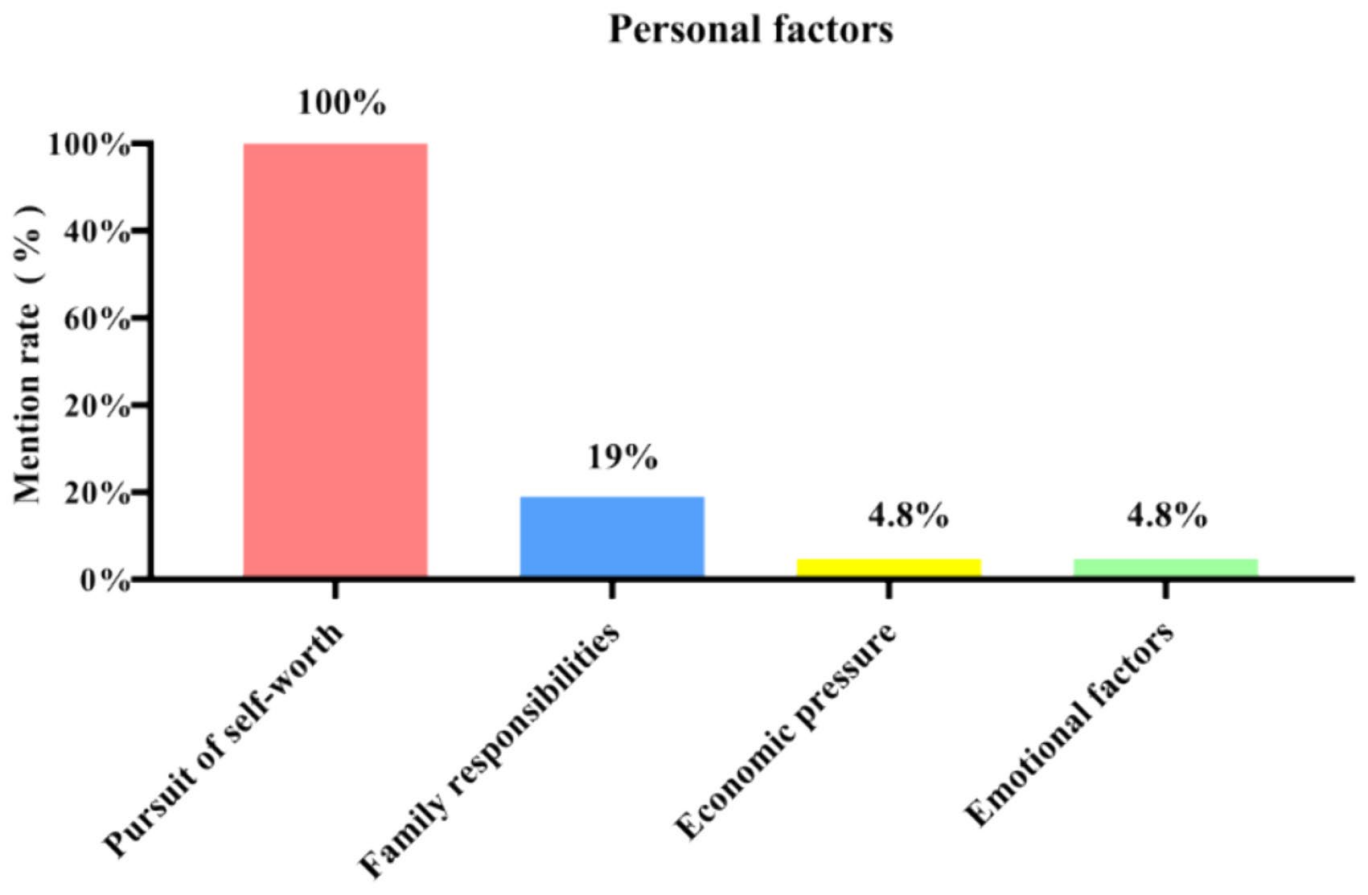
Mentions of the four sub-categories under the personal factor among the 21 in-depth interview respondents.

From Figure 4, it can be concluded that the pursuit of self-worth is mentioned as 100% of the personal factors showing that high-level talents are particularly focused on this pursuit which includes many aspects. Young doctors who have just joined the profession are highly educated, have strong personalities, are free-spirited, face up to injustices and grievances, complain when they are unfairly treated, and seek to achieve self-fulfillment by changing their work environment to one of greater possibilities of development (14).

### Category 2: hospital culture

It can be concluded that when discussing hospital culture, 100% of the departed employees mentioned work atmosphere, and 71.4% mentioned work pressure.

**Figure 5:**
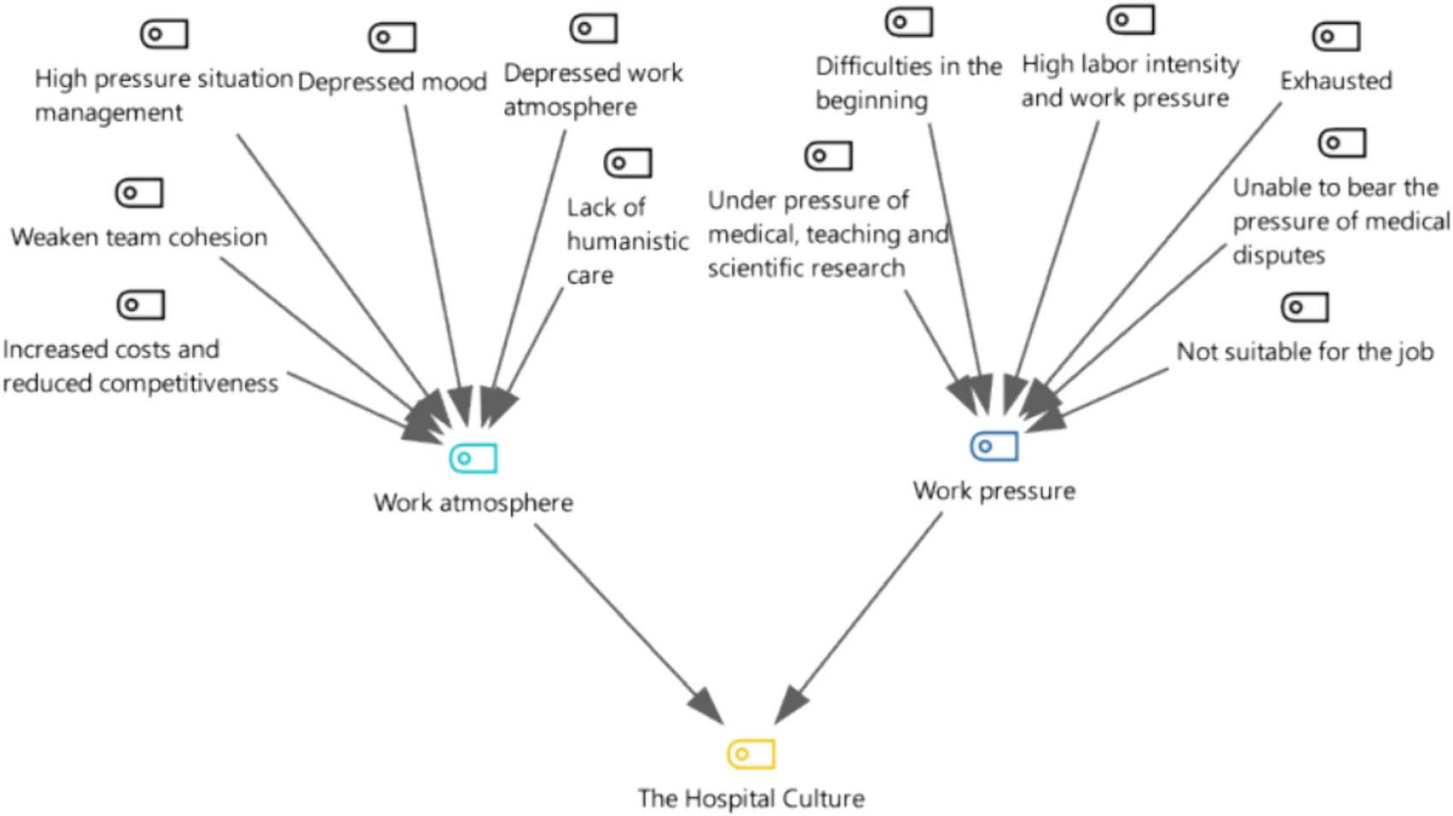
Three-level coding chart for the hospital culture category.

**Figure 6:**
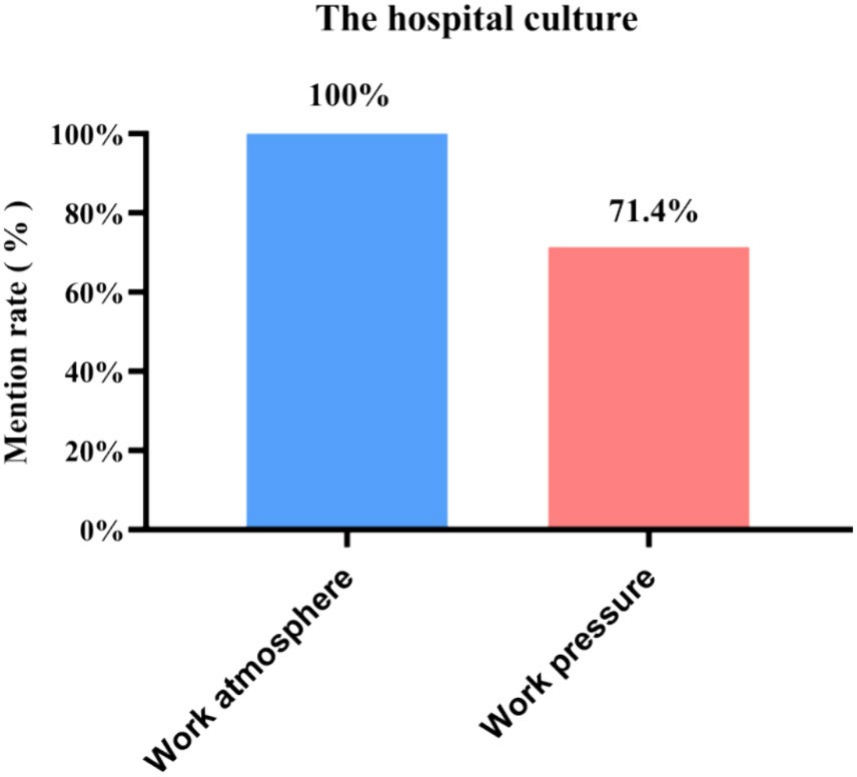
Mentions of the two sub-categories under hospital culture among the 21 in-depth interview respondents.

Respondents perceived the working atmosphere in the hospital as oppressive. The ability of employees to express their opinions and participate freely and forcefully in the management decision-making process and the ability of the organization to create a good organizational climate for decision-making activities are important prerequisites for reducing employees’ willingness to leave (7, 15). The work atmosphere affects employees’ creative energy and commitment to work. A depressing work atmosphere can cause a constant brain drain and affect the stability of the workforce (Figures 5, 6).

### Category 3: hospital system

**Figure 7:**
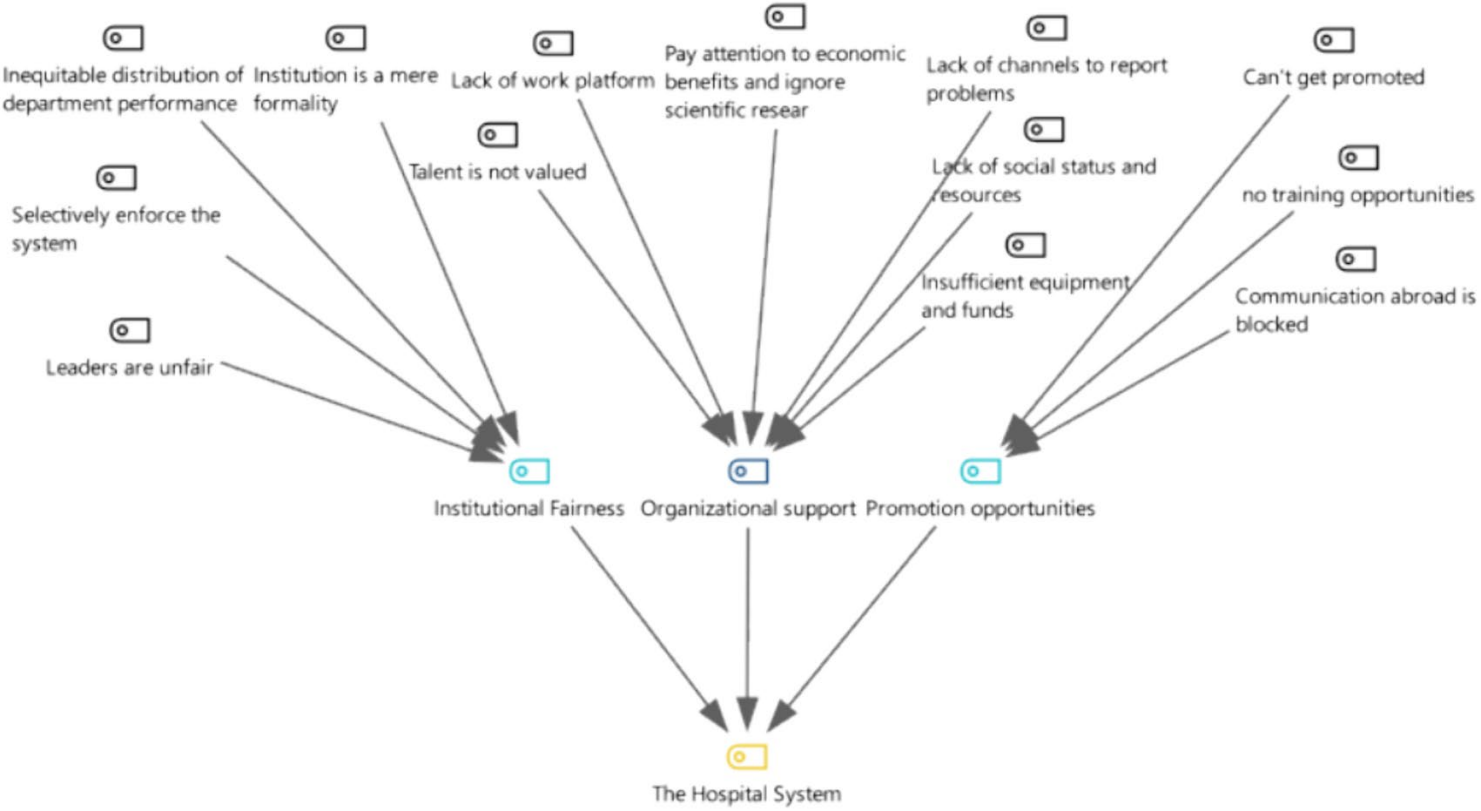
Three-level coding chart for the hospital system category.

**Figure 8:**
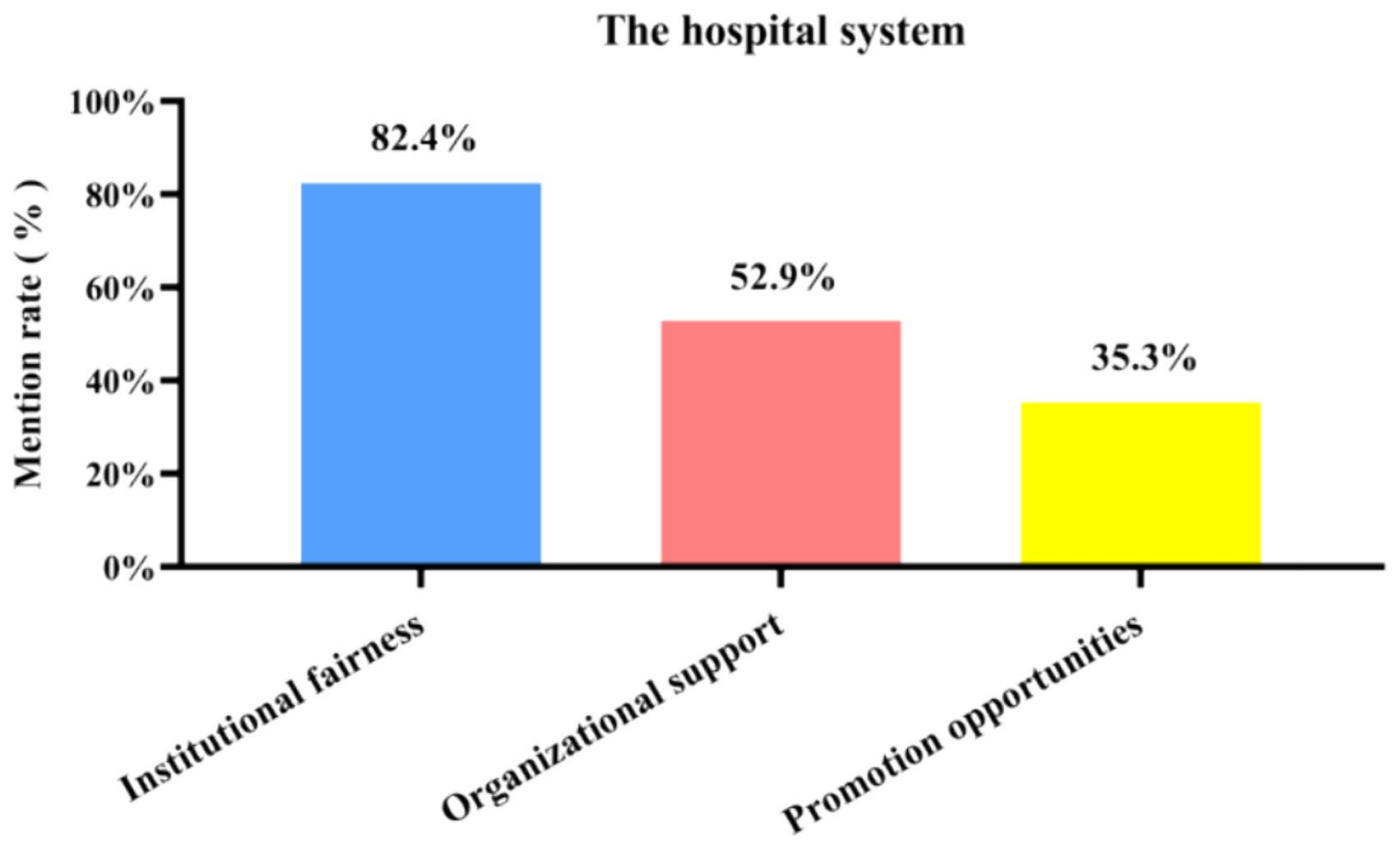
Mentions of the three sub-categories under hospital system among the 21 in-depth interview respondents.

From Figures 7, 8 it can be seen that, according to the interviewees, the hospital system is mainly influenced by three sub-categories: system fairness, organizational support and promotion opportunities. Of the 21 departed employees who were interviewed in depth, 82.4% mentioned system fairness, 52.9% mentioned organizational support, and 35.3% mentioned promotion opportunities as the main sub-categories within the hospital system.

A lack of fair trust in an organization can led to lower job satisfaction and willingness to leave (16). Respondents mentioned that the implementation of a system in the case hospital is just a formality, which hurts the relationship between the hospital and employees, and is one of the important reasons for the brain drain.

### Category 4: social environment

Departures are closely related to the number of existing firms outside the organization and that the more competitors and opportunities there are in the industry, the more likely it is for employees to leave (5, 17). Industry competition and the introduction of national policies will affect the loss of talent to a certain extent.

**Figure 9:**
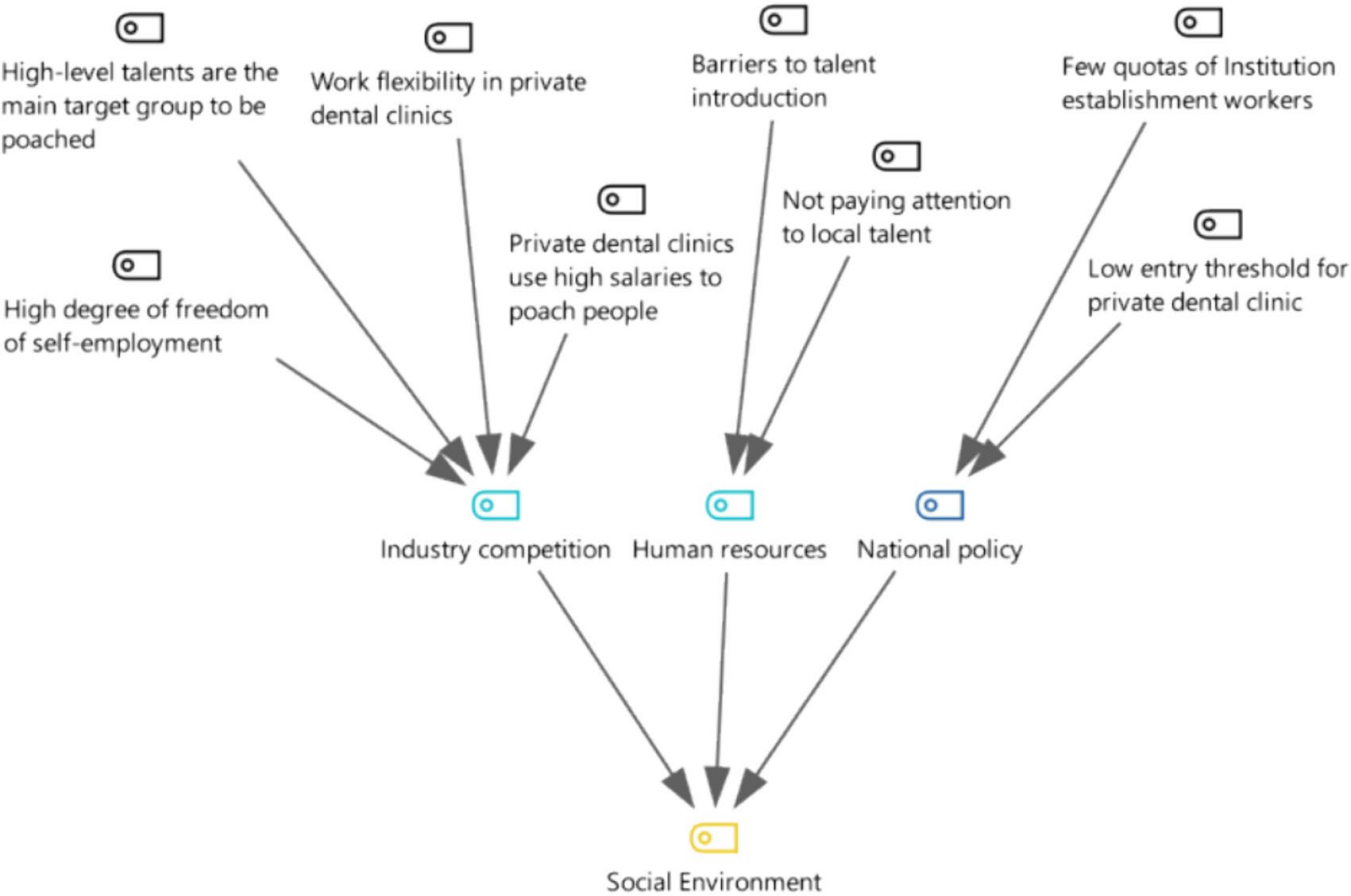
Three-level coding chart for the social environment category.

**Figure 10:**
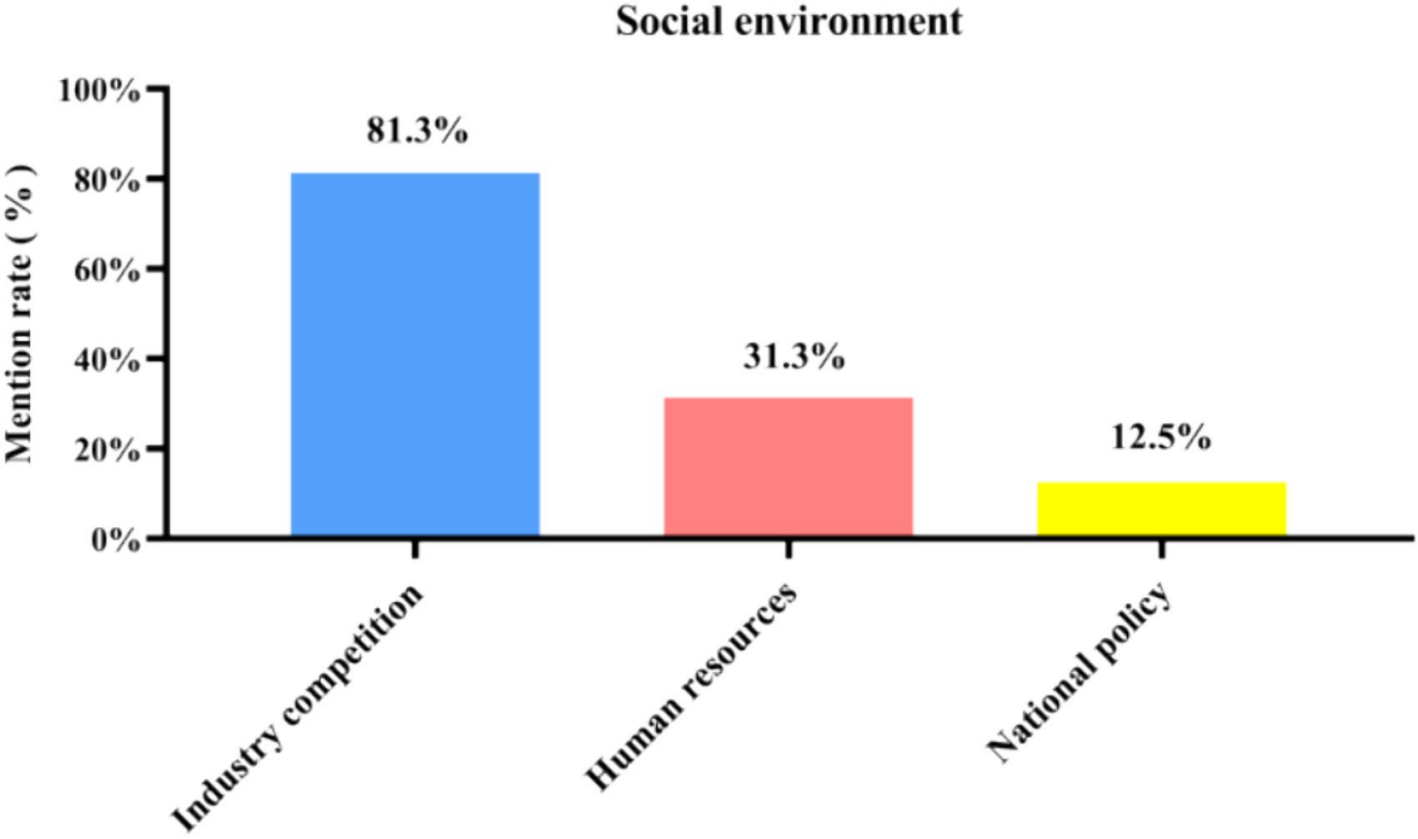
Mentions of the three sub-categories under social environment among the 21 in-depth interview respondents.

From Figures 9, 10, it can be concluded that the main factors affecting the departure of high-level talents in the social environment are industry competition (81.3% of mentions), human resources (31.3%), and national policies (12.5%). Industry competition means the threat that private dental clinics pose to public dental hospitals. On the one hand, private dental clinics have better working conditions; on the other hand, they tend to use high salaries to poach high-level talents from public dental hospitals. The sub-category “human resources” indicates that, according to the interviewees, the hospital did not pay attention to local talents and set obstacles to bringing in new talents. In turn, interviewees also mentioned that national policies contributed to the social environment by restricting quotas for tenured employees at public hospitals and lowering the requirements for the establishment of private dental clinics.

### Category 5: leadership team

Ineffective leadership can lead to job dissatisfaction and negativity, which in turn can spur turnover. In the medical sector, the management style is also directly related to patient outcomes, regardless of work experience (18). The mission of managers is to develop employees and build effective teams, rather than imposing their work on themselves, overstepping their responsibilities, and participating in everything (19). Instead, they should motivate employees to participate in the management of enterprise objectives so that employees can be most motivated toward reaching the same goal (20).

**Figure 11:**
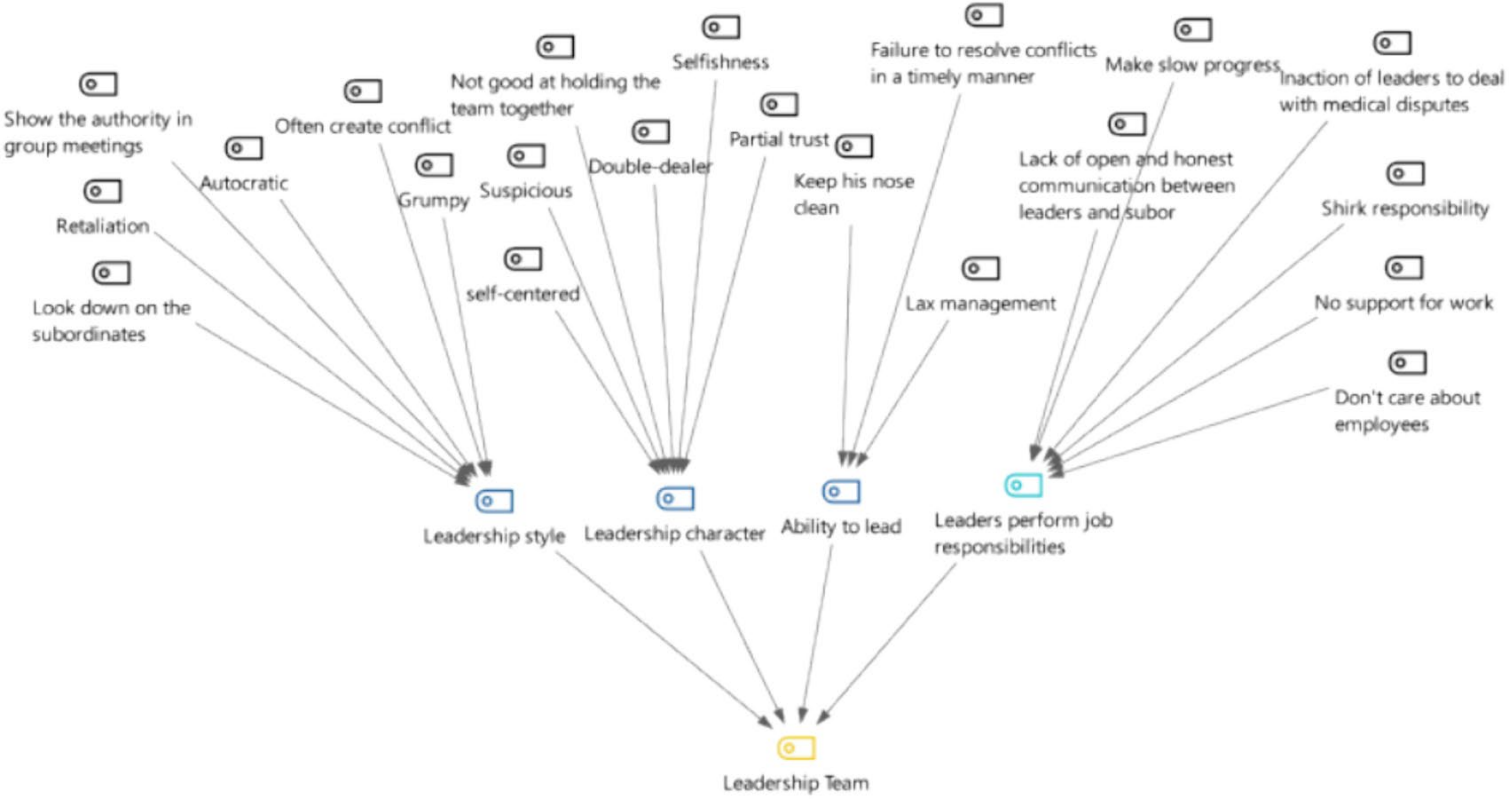
Three-level coding chart for the leadership team category.

**Figure 12:**
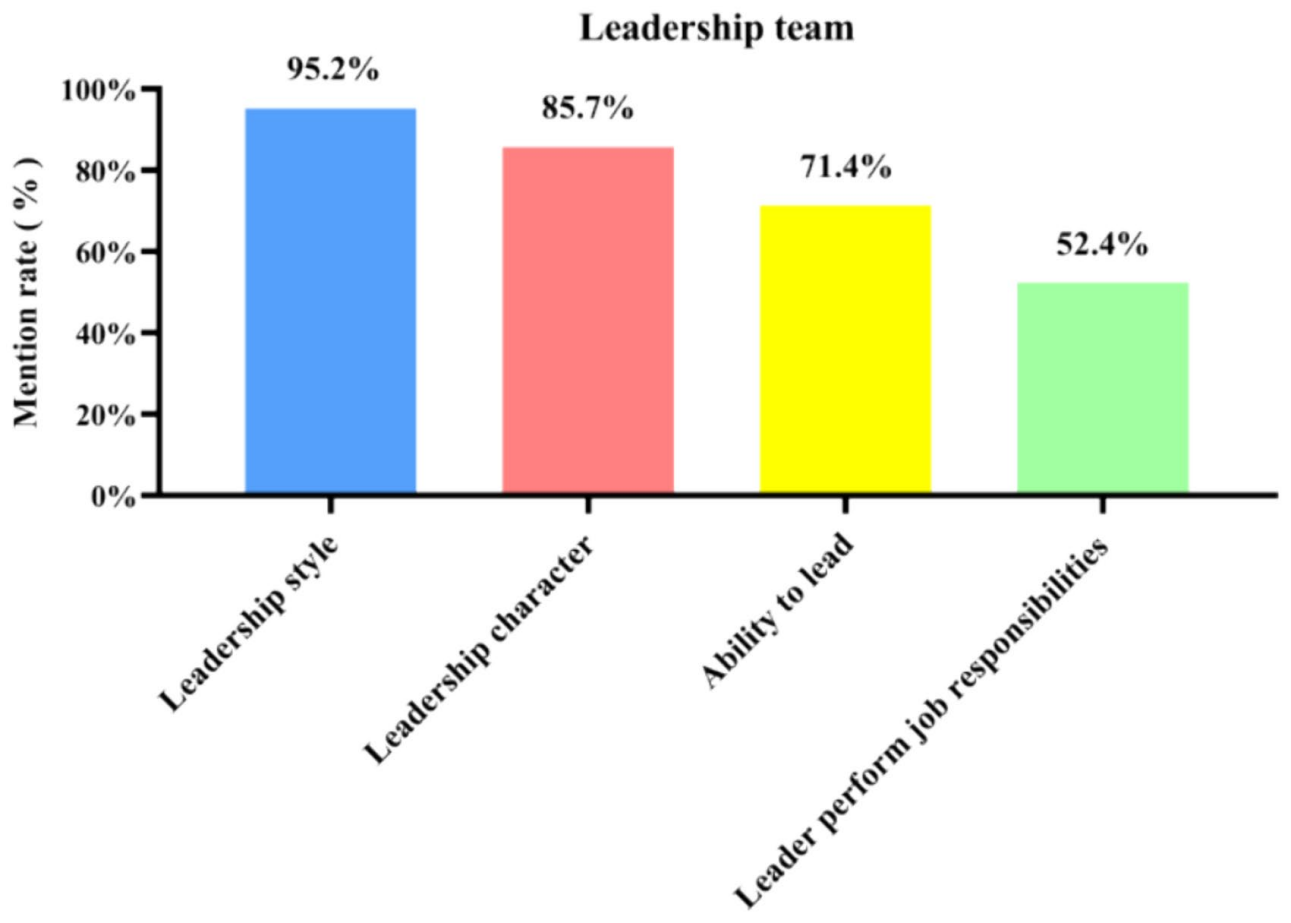
Mentions of the four sub-categories under the leadership team among the 21 in-depth interview respondents.

From Figures 11, 12, it can be concluded that, according to the interviewees, the influencing factors in the leadership team are leadership style (95.2% of mentions), leadership character (85.7%), leader’s ability (71.4%), and leader’s performance of job responsibilities (52.4%).

### Category 6: CPC’s leadership system

**Figure 13:**
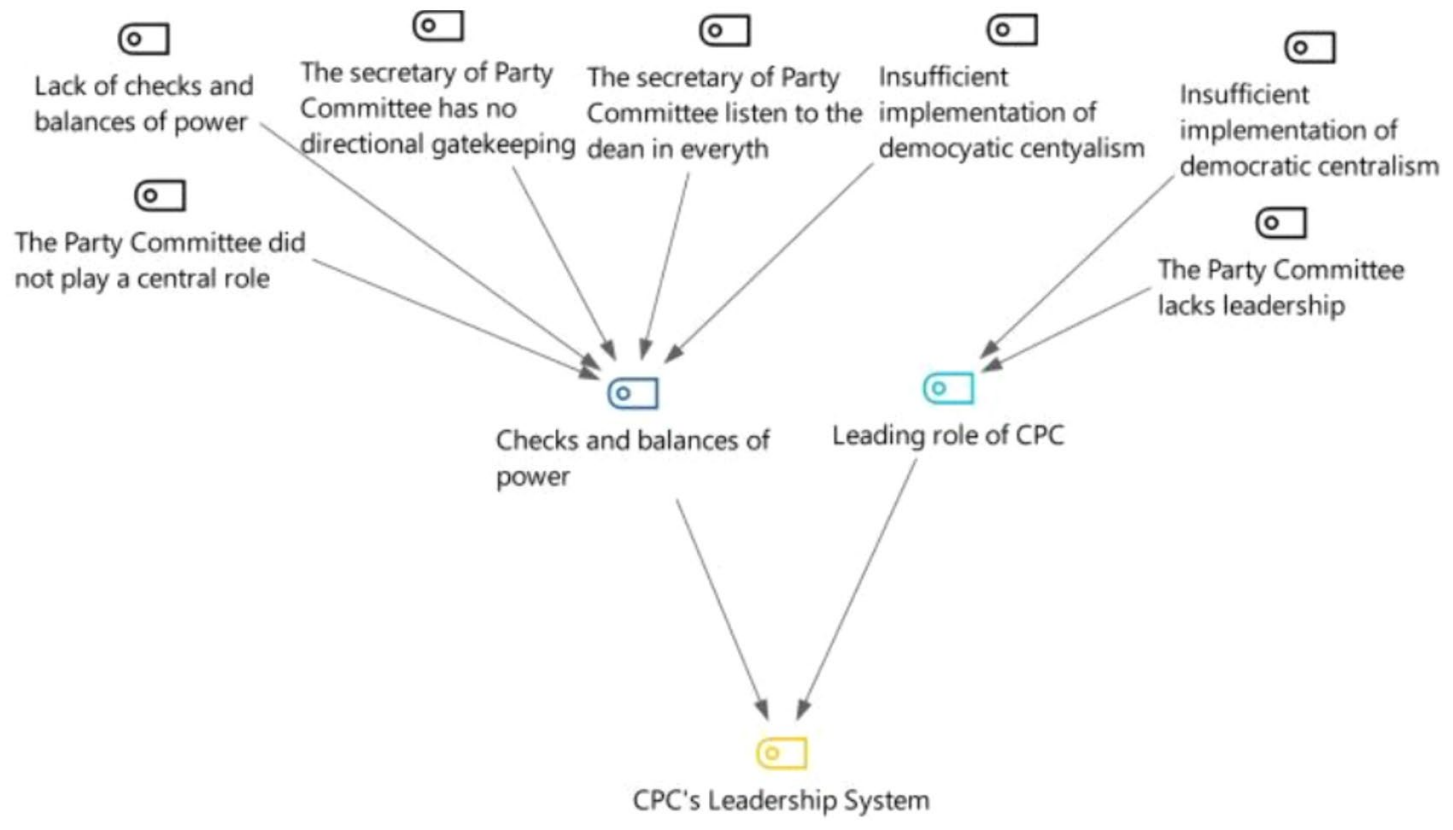
Three-level coding chart for the CPC’s leadership system category.

**Figure 14:**
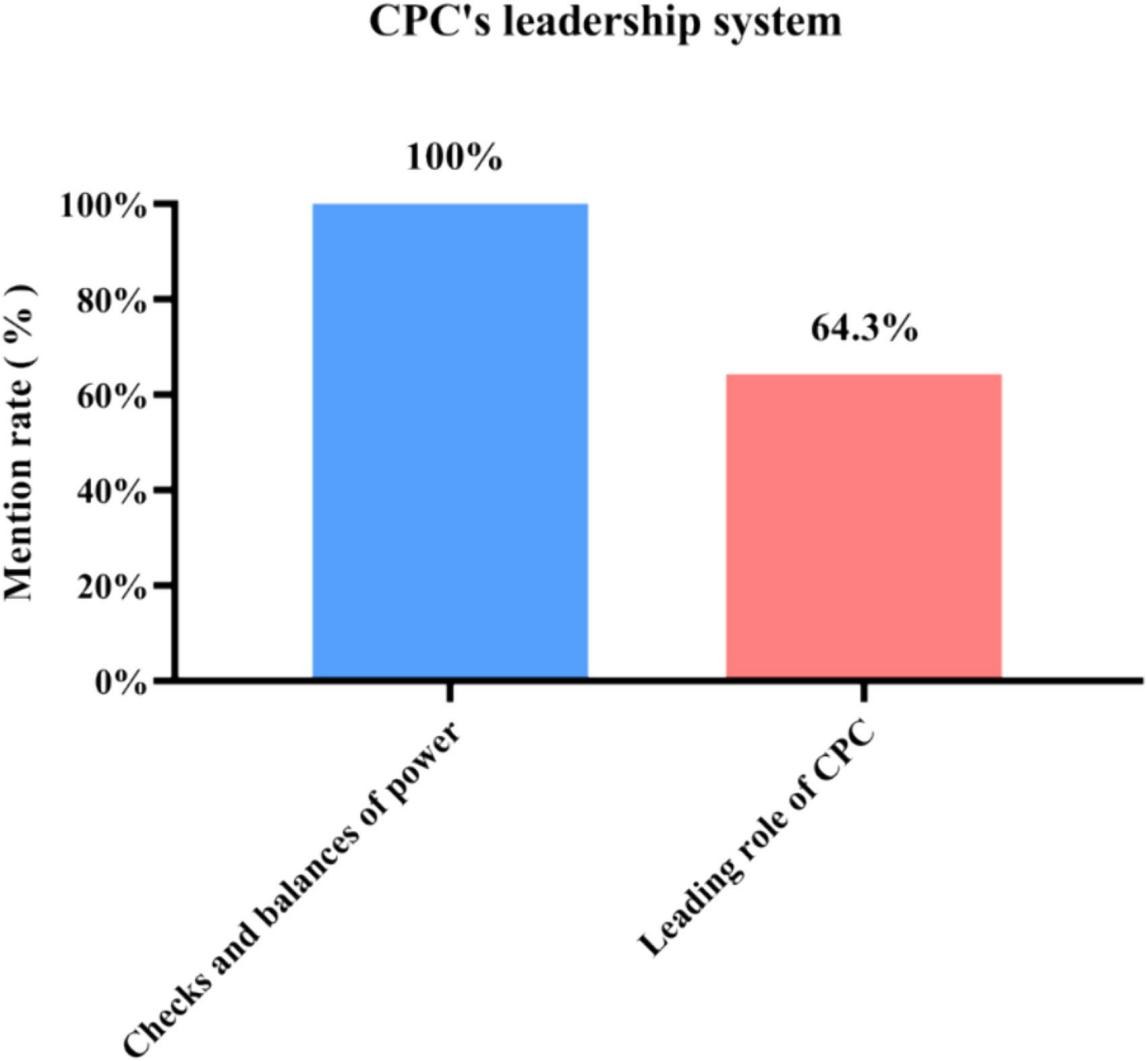
Mentions of the two sub-categories under CPC’s leadership system among the 21 in-depth interview respondents.

From Figures 13, 14, it can be concluded that the influencing factors in the party’s leadership system are power checks and balances and the leading role of CPC. In the interviews, “checks and balances” were mentioned 100%, and the leading role of CPC was mentioned 64.3%. In terms of the leading role of the CPC, respondents mainly cited the lack of leadership of the Party committee and the disunity of the leadership teams in the hospital studied. In terms of checks and balances of power, the interviewees mentioned that the Secretary of the Party committee followed all the instructions from the President and did not play the role of gatekeeper and supervisor. The dual management system did not follow the principle of democratic centralism and lacked checks and balances of power.

## Discussion

The importance of different categories was compared and explored and finally divided into six categories (aggregate dimensions): “personal factors,” “hospital culture,” “hospital system,” “social environment,” “leadership team,” and “CPC leadership system.” These categories were derived from the relationships among the factors mentioned by the interviewees that first generated key sub-categories and, after further coding, gave way to the main categories above.

**Figure 15:**
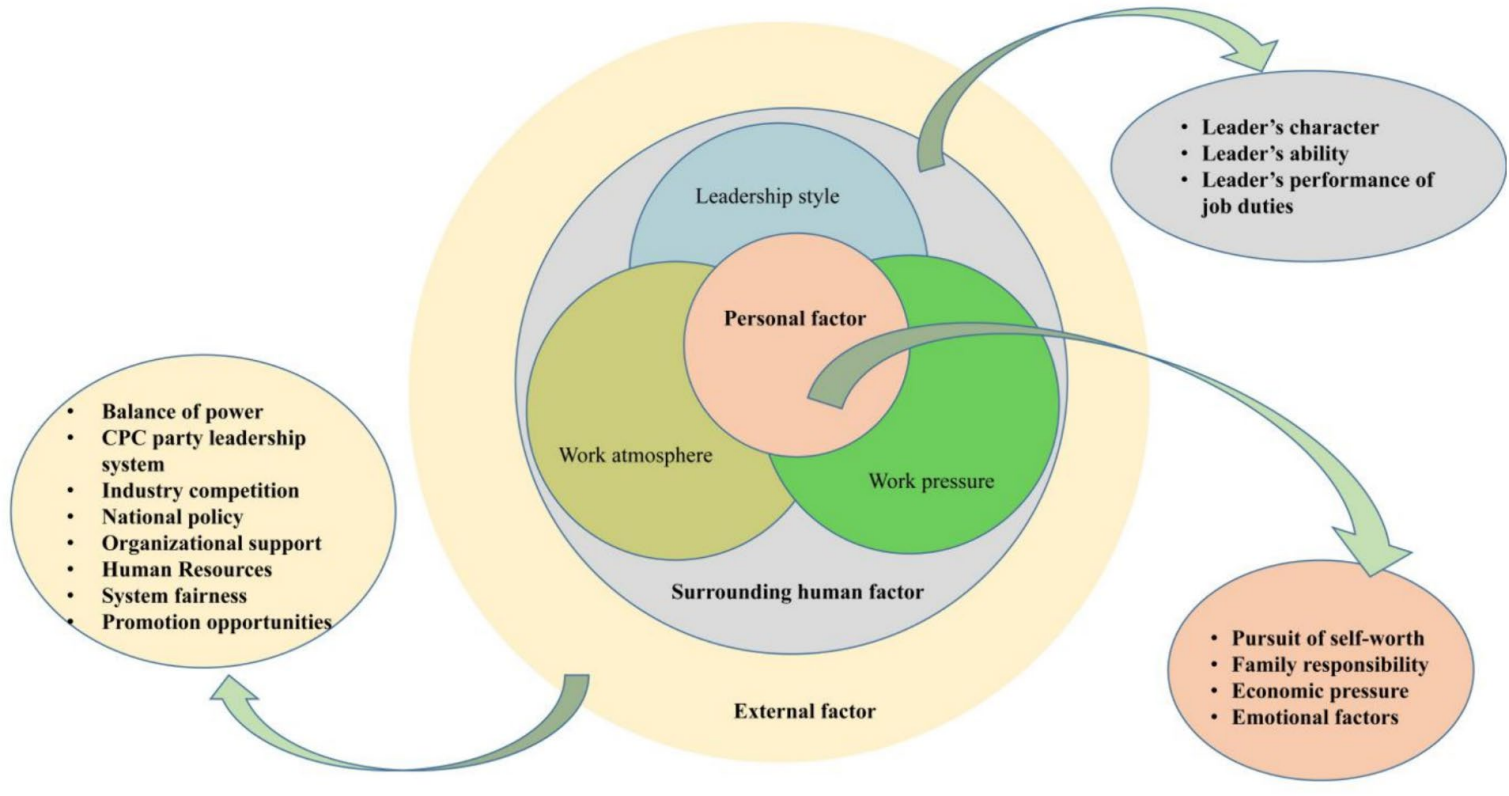
Theoretical model diagram.

The relationship among these categories was analyzed as the theoretical basis for this study and divided into the following three parts (Figure 15).

- External factors are represented in the external ring of the figure and relate to the system and environment (national policy and industry environment); the Party leadership system (checks and balances of power and leadership); and the social environment (fairness and organizational support).
- Surrounding factors are represented in the middle ring and concern the leadership team and hospital culture, namely work pressure and work atmosphere.
- Personal factors are shown in the internal ring and include the pursuit of self-worth, family responsibility as well as other personal and economic factors that interact with one another.

External factors directly influence surrounding factors, which in turn affect the hospital system. The hospital system and the social environment also directly affect personal factors, which interact with the leadership of the Party system. If there is a mismatch between the core of personal factors and the feedback from other factors, this will directly lead to personnel turnover.

In the above 18 categories, the mentioned rate of power checks and balances in the interview is 100%, the mentioned rate of leadership style is 95.2%, the mentioned rate of system fairness is 82.4%, the mentioned rate of work pressure is 100%, and the mentioned rate of work atmosphere is 71.4%. As a key factor in the model, the relationship between key factors will be explored by combining the expression relationship between relevant factors in relevant literature and interview materials, and the relationship structure of demission factors. In this study, through in-depth communication with the respondents, it can be concluded that the imbalance of power in the dual management system leads to the overbearing personal style of the leaders, which affected the working atmosphere of the hospital and led to great pressure on the employees and to the high turnover of talents. This suggests that there is a close relationship between the right balance and leadership style, between leadership style and job stress, between leadership style and system fairness, between work stress and achieving personal self-worth, and between the working atmosphere and employee turnover. Through detailed analysis, the relationship among different factors is hereafter discussed.

### The balance of power and leadership style

The “checks and balances of power” category is layered down from five initial concepts in the 21 in-depth interviews represented by statements such as: “the Party secretary has no checks on the power of the president,” “the Party secretary has not used his power well,” “the Party committee failed to play a central role,” “the principle of democratic centralism was not implemented,” and “the power of checks and balances is lacking.” The idea of checks and balances of power was born in the works of the Greek historian Polybius (c. 200 – c. 118 BC), evolved into the theory of the “separation of powers” in the Middle Ages, continued through the Renaissance, and into the Western bourgeois revolution, and played an important role in promoting the progress of human political civilization. Excessive concentration of power will inevitably lead to the formation of absolute power lacking necessary supervision and restriction, which makes the abuse of power uncontrolled, and the internal governance easily taken over by individuals or a few people (21). The system of checks and balances avoids power concentration and abuse and fully reflects the existence of harmonious leadership behavior in a dual management system.

Some scholars have raised the question of whether the checks and balances of power can be used in the case of senior executives to restrain the power of the CEO and prevent the execution of risky projects for personal interests that may damage corporate value. For example, the stronger the balance of power of the management, the more it can prevent the abuse of power of the chairman or general manager and further optimize the investment decisions of enterprises. There is a direct causal relationship between the balance of power as a system and the leader’s behavior, and the imbalance of power is more likely to lead to the abuse of power.

The category “leadership style,” also frequently mentioned by respondents, refers to the unique and habituated behavior formed by leaders after long-term leadership practice and is closely related to the ruling environment of leadership. Besides internal factors, the formation of a leadership style is also related to the institutional environment.

In China, the president’s responsibility system under the leadership of the Party committee is implemented as a dual management system, and the authority of the president and the secretary of the Party committee is divided. The president’s responsibility system means that not all the power is concentrated in the president’s hands. The Party organization is the “core of leadership” and the one that discusses and makes decisions on major issues. Once the leadership of the Party organization is weak, the power of the president will expand rapidly creating internal deficiencies. Then, the president’s responsibility system will become “the president’s system” and the leadership style of the president will be more and more arbitrary and overbearing. Therefore, power imbalances can lead to changes in leadership styles.

### Leadership style and work stress

According to the interviewees, the leadership style in the case hospital at the time of their tenure is one of the 18 sub-categories extracted from the 21 in-depth interviews. Leadership has different standards and characteristics including the abusive leadership style that matches the concepts raised by the interviewees (22). As they described, in abusive leadership styles, employees are often subjected to malicious verbal attacks and non-verbal behavior from their leaders. It is a typical negative leadership behavior, which brings a series of negative effects, including a significant negative impact on employees’ daily work and family life. On this basis, emotional management and emotional guidance to reduce employees’ work stress are proposed to solve the adverse effects caused by abusive leadership styles (23).

As an important factor of organizational characteristics and the main source of employees’ emotions, in the process of getting along with their subordinates, too strong a leadership style as well as the arbitrary exercise of power through aggressive attitudes to impose their will on employees, will affect their emotions and work enthusiasm. In this environment, employees are always in a tense state and the communication between superiors and subordinates is not smooth leading to high work pressure. Sincere leadership style and empathy are moderating factors in the effect of work stress on work quality, and help to alleviate negative impacts.

### Leadership style and institutional fairness

Institutional fairness is a sub-category that emerged from the 21 in-depth interviews and contributes to the aggregate dimension (category) designated as “hospital system.” We understand it as a kind of organizational justice from the perspective of individuals. Organizational justice refers to the perception of fairness created by rules, regulations, basic policies, and effective measures in the organizational structure of a social group. To some extent, ensuring organizational justice is the result that all social organizations should strive to achieve. Specifically, the building of organizational justice comes from three sources: institutional fairness, distributional fairness, and communication fairness. The leader’s impartiality, recognition, attention, and respect for subordinates can enable employees to maximize their release of energy.

The hospital system is the code of conduct of all staff and the embodiment of hospital management philosophy, thoughts, and mode, which will be related to the rise and fall of hospital survival. Every regulation needs to be established based on equal treatment, everyone is equal before the system, which is conducive to the healthy development of the hospital. If the leadership style is dictatorial, it will destroy the fairness of the system to safeguard the interests of one individual or small groups. An abusive leadership style will destroy communication fairness and, if in the process of work communication, the leader abuses the authority role to put pressure on the subordinate, he or she does not give basic respect to the subordinate. Likewise, in the process of information transmission, if the information is not balanced enough and is selectively delivered to specific groups, this will affect the employees’ sense of fairness. The above concepts reflect the unfair implementation of the main leadership system in the case hospital, which is conducive to a lack of fairness in the system.

### Work stress and personal self-worth

Work stress is a sub-category resulting from 10 initial concepts in the 21 in-depth interviews: “profession and post do not match,” “working the night shift is difficult,” “entrepreneurial hardships,” “labor intensity and working pressure,” “medical treatment, teaching, scientific research, and comprehensive development create high pressure,” “poor conditions,” “occupational identity is not strong,” “exhaustion of body and mind,” “cannot adapt to work,” and “difficult to face medical disputes under pressure.” As employees pay more attention to their career success and the realization of their value, related studies have shown that work stress is negatively correlated with professional identity and that individual components of work stress such as organizational management, career interest, and career development are also negatively correlated with professional identity (23).

The rationality of the organizational system has a great influence on the professional identity and turnover intention of clinicians and unreasonable management often reduces their job satisfaction. In addition, an incomplete cognition of a career, vague direction, or lack of interest in career development will lead to low professional identity. This was particularly sensed during the interviews when one department director said that he felt that going to work was like going to prison, an everyday struggle under huge working pressure. He had to leave to get out of trouble. Those employees interviewed both in the exit and post-facto interviews confessed to having faced great work pressure, and physical and mental exhaustion. Under the dual role of work pressure and psychological intolerance, employees lacked a sense of belonging, felt lonely and dissatisfied, and lacked security, all of which inevitably produced corresponding value judgments that manifested in new career choices.

### The working atmosphere and talent turnover

The category working atmosphere emerges from 14 initial concepts in the 21 in-depth interviews: “creates feelings of loss,” “interpersonal complexity,” “weakens team cohesion,” “shakes people up,” “affecting the stability of the team,” “poor image of the hospital,” “increasing costs,” “reduced competitiveness,” “depressed working atmosphere,” “depressed mood,” “restrictions,” “lack of humanistic care,” “lack of cohesion and centripetal force,” “marginalized,” “forced resignation,” and “high-pressure situation management.” To a large extent, work atmosphere refers to the psychological environment of organization members, which is the consensus generated by most people after internalizing the same type of environment. The work atmosphere is a hidden soft power that can make people happy or unhappy and affect work efficiency. A bad working atmosphere makes people feel depressed, and emotionally exhausted, with a lack of belonging those results in job burnout, turnover intention, and eventually in turnover.

When members trust their team, they will have positive expectations about the attitude and behavior of others in the team, and then feel secure (24). On the contrary, if team members generally have low trust in the team, they will become depressed due to a lack of security. When the work atmosphere is negative, employee mentality and team spirit are inevitably passive. The interviewees fully reflected the dull atmosphere in the hospital: they had no sense of belonging and pride, lost confidence in the future, and finally chose to leave.

From these five relationships, it is concluded that: power checks and balances affect the leadership style, the leadership style affects leadership behavior, and leadership behavior affects employee turnover. The more imbalance of power, the more prominent the personal style of the leader, and if the personal style is tyrannical, the greater the impact on employee turnover.

The balance of power in the case hospital affected leadership style, and the leadership style affected the leadership behavior, which in turn affected employee turnover. In a dual management system, the more unbalanced the power, the more prominent a leader’s style is. If the personal style is bossy, the greater the impact on employee turnover.

Based on the Chinese context, in a dual management system characterized by the general responsibility of the director under the leadership of the Party committee, there is a great possibility of power division between the Party affairs and administrative leaders. Checks and balances refer to the existence of forces within or outside public political power that counterbalance the subject of power. Such forces often determine the leadership team’s ability to take the overall situation and seek development. If there is a power imbalance, the leadership team may have a situation in which individual behavior determines the development of the organization. In this case, the personal style of the leader directly determined the institutional fairness, hospital culture, and working atmosphere of the hospital, and these factors directly affect the overall demission of talented hospital staff.

The case of the hospital studied reflects the conclusion of the above empirical research. Because the Party committee did not play a central role, the power imbalance between the Party secretary and the president occurred affecting the hospital culture and leading to brain drain.

## Conclusion and suggestions

Upon the results of previous research and the analysis of this thesis, it can be concluded that the most important factors affecting the demission of employees are organizational factors. In the case hospital, only by optimizing the related system of the organizational structure, can we further reduce the demission rate. Although there are various reasons for the problem, both the data and the literature point out the failure of checks and balances of power which directly leads to feelings of insecurity and chaos (25). The key to an effective balance of power lies in the separation of powers in a legitimate way, to balance power with power, and safeguard rather than damage the legitimate interests of the people.

In organizations in China, the dual management system, comprising the President’s responsibility system under the leadership of the Party Committee, is meant to prevent the abuse of power sustained by a solid ideological and moral defense line. The most fundamental ideological requirement for the realization of checks and balances of power is to adhere to the concept that “power is granted by the people and used by the people.”

## Data Availability

The original contributions presented in the study are included in the article/supplementary material, further inquiries can be directed to the corresponding authors.

## References

[1] Zhu MH. The causes and countermeasures of tension between doctors and patients. Fam Health. (2019) 16:36.

[2] Zheng XY Yuan QF. Exploration of talent attraction strategies in public hospitals under the new situation. Chin Health Stand Manag. (2022) 13:49–53.

[3] Tian S. M. Fan Y. Liu Y. L. Wu Y. (2014). “Discussion on the model of coordinated development of medical treatment, teaching and research in affiliated hospitals” in *National Symposium on “SCI Papers and Healthcare, Teaching and Learning”*.

[4] Griffeth RW Hom PW. Retaining Valued Employees. Thousand Oaks: Sage (2001).

[5] March JG Simon HA. Organizations. New York: Wiley. (1958).

[6] Mobley WH. Intermediate linkages in the relationship between job satisfaction and employee turnover. J Appl Psychol. (1977) 62:237–40. doi: 10.1037/0021-9010.62.2.237

[7] Lee TY Tzeng. Effects of a preceptorship programme on turnover rate, cost, quality, and professional development. J Clin Nurs. (2009) 18:1217–25. doi: 10.1111/j.1365-2702.2008.02662.x 19320789

[8] Giske T Artinian B. A personal experience of working with classical grounded theory: from beginner to experienced grounded theorist. Int J Qual Methods. (2007) 6:67–80. doi: 10.1177/160940690700600405

[9] GOCPCCC. (2018). General office of the CPC central committee: Opinions on strengthening party building in public hospitals.

[10] Hennink MM Kaiser BN Weber MB. What influences saturation? Estimating sample sizes in focus group research. Qual Health Res. (2019) 29:1483–96. doi: 10.1177/1049732318821692 30628545 PMC6635912

[11] Rijnsoever FJV. Saturation: a simulation and guidelines for sample sizes in qualitative research. PLoS One. (2017) 12:e0181689. doi: 10.1371/journal.pone.0181689, PMID: 28746358 PMC5528901

[12] Pan S Yao X Huang Y. The number of the interviewees in qualitative study: is it the issue of" representing a population" or "representing what”? ——methodological meaning of the “maximum difference and information saturation sampling strategy”. Soc Sci Res. (2010) 8:108–15.

[13] Zhou D. Alfred Adler: a landmark psychologist who promoted human spiritual development. J Inner Mongolia Univ Nat. (2005) 31:107–9.

[14] Chaacha TD Botha E. Factors influencing intention to leave of younger employees in an academic institution. SA J Hum Resour Manag. (2021) 19:1683–7584.

[15] Halfer GS. The organizational impact of a new graduate pediatric nurse mentoring program. Nurs Econ. (2008) 4:243–9. 18777973

[16] Kim SJ Tam L. Determinants of employee turnover intention: understanding the roles of organizational justice, supervisory justice, authoritarian organizational culture and organization-employee relationship quality. Corp Commun Int J. (2017) 22:308–28. doi: 10.1108/CCIJ-11-2016-0074

[17] Price JL. Reflections on the determinants of voluntary turnover. Int J Manpow. (2001) 22:600–24. doi: 10.1108/EUM0000000006233

[18] Danae S Aggelos L Athina P Dimitra SP Michail M Evridiki P. Importance of leadership style towards quality-of-care measures in healthcare settings: a systematic review. Health. (2017) 5:73. doi: 10.3390/healthcare5040073 PMC5746707 29036901

[19] Fan D. Replicable Leadership. 1st ed. Beijing: CITIC Press Group (2018).

[20] Inamori K. Amoeba management. Mod State Own Enterp Res. (2011) 11:42–7.

[21] Ren JT Tong DZ Zhang ZX Ren JM Gong T Tang YL. Supervision of power in the context of people's democracy in the whole process. J Guangzhou Univ Soc Sci. (2022) 21:31.

[22] Walsh MM Arnold KA. The bright and dark sides of employee mindfulness: leadership style and employee well-being. Stress Health. (2020) 36:287–98. doi: 10.1002/smi.2926 31957962

[23] Menglin Z. Study of relationship between professional identity and work stress, coping style of doctors in a third-grade class-a hospital in Qiqihar city. Med Soc. (2019) 32:107–110. doi: 10.13723/j.yxysh.2019.06.029

[24] Zhu F Wang L Yu M Müller R Sun X. Transformational leadership and project team members' silence: the mediating role of feeling trusted. Int J Manag Proj Bus. (2019) 12:845–68. doi: 10.1108/IJMPB-04-2018-0090

[25] Zhou X. (2010). Studies in the politics of the Roman Republic. Doctoral dissertation. Jilin University.

